# Dependence of mitochondrial dysfunction in peripheral blood mononuclear cells on cervicocephalic atherosclerotic burden in acute ischemic stroke

**DOI:** 10.3389/ebm.2025.10624

**Published:** 2025-07-01

**Authors:** Xiaoxi Zhao, Yi Yang, Xiangying Du, Luguang Li, Chengbei Hou, Yanning Cai, Xin Ma

**Affiliations:** ^1^ Department of Neurology, Xuanwu Hospital, Capital Medical University, Beijing, China; ^2^ National Clinical Research Center for Geriatric Disorders, Beijing, China; ^3^ Department of Radiology, Xuanwu Hospital, Capital Medical University, Beijing, China; ^4^ Center for Evidence-Based Medicine, Xuanwu Hospital, Capital Medical University, Beijing, China; ^5^ Department of Neurobiology, Xuanwu Hospital, Capital Medical University, Beijing, China; ^6^ Department of Clinical Biobank, Xuanwu Hospital, Capital Medical University, Beijing, China

**Keywords:** atherosclerosis, mitochondrial deoxyribonucleic acid copy number, reactive oxygen species, peripheral blood mononuclear cells, acute ischemic stroke

## Abstract

As an inflammatory disease, atherosclerosis is associated with acute ischemic stroke (AIS), but its early identification and intervention efficacy remain suboptimal. A new research direction may be to explore peripheral atherosclerotic biomarkers from the perspective of mitochondrial dysfunction, which can induce inflammatory cell activation. Moreover, the degree of overall cervicocephalic atherosclerosis (namely, atherosclerotic burden) is more closely related to AIS prognosis than local atherosclerotic lesions. Therefore, this study investigated the relationship between mitochondrial dysfunction in peripheral blood mononuclear cells (PBMCs), including monocytes and lymphocytes, and overall cervicocephalic atherosclerotic burden and AIS outcome. Patients with AIS and cervicocephalic atherosclerosis were enrolled and followed up for 90 days. The reactive oxygen species (ROS) and the mitochondrial deoxyribonucleic acid copy number (mtDNA-CN) in PBMCs were measured respectively through a fluorescence probe and a droplet digital polymerase chain reaction to evaluate mitochondrial function. The overall intracranial and cervical atherosclerotic burden (ICAB) was quantified by summing up the atherosclerosis degree points in each arterial segment as assessed by computed tomography angiography. A modified Rankin Scale (mRS) score >2 was considered a 90-day unfavorable functional outcome. Five (4.9%) of the 103 patients with AIS were lost to follow-up. mtDNA-CN [adjusted β = −0.099, 95% confidence intervals (CIs) = −0.153 ∼ −0.044, *p* < 0.001] and ROS content (adjusted β = 1.275, 95%CI = 0.885 ∼ 1.665, *p* < 0.001) were correlated with ICAB. The risk of a 90-day unfavorable functional outcome increased with higher ROS content [adjusted odds ratio (OR) = 1.523, 95%CI = 1.172 ∼ 1.981, *p* = 0.002] and decreased with higher mtDNA-CN (adjusted OR = 0.911, 95%CI = 0.850 ∼ 0.976, *p* = 0.008). PBMC mitochondrial dysfunction was found to be independently associated with extensive and severe cervicocephalic atherosclerosis and a 90-day unfavorable functional outcome in patients with AIS, which may provide a novel approach to improving the early identification and risk stratification of cervicocephalic atherosclerosis, along with the prediction of the outcome of atherosclerotic AIS.

## Impact statement

As a chronic progressive inflammatory disease, atherosclerosis is a primary etiology of acute ischemic stroke (AIS), but its early identification and intervention efficacy remain suboptimal. It may be a breakthrough to explore peripheral atherosclerotic biomarkers from the perspective of inflammatory cells mitochondrial dysfunction. Moreover, the overall cervicocephalic atherosclerosis degree is more closely related to AIS prognosis than the presence of local atherosclerotic lesion. Thus, we investigated the relationship between peripheral blood mononuclear cells (PBMC) mitochondrial dysfunction (mitochondrial deoxyribonucleic acid copy number reduction and reactive oxygen species overexpression) and intracranial and cervical atherosclerotic burden (ICAB). PBMC mitochondrial dysfunction was found to be independently associated with extensive and severe cervicocephalic atherosclerosis (high ICAB) and poor short-term functional outcome in AIS patients with cervicocephalic atherosclerosis. These findings may provide a feasible new approach to improve the identification and risk stratification of total cervicocephalic atherosclerosis degree and functional prognosis prediction of atherosclerotic AIS patients.

## Introduction

Atherosclerosis is the primary cause of acute ischemic stroke (AIS) [1] and is closely correlated with AIS prognosis [2, 3], necessitating the early identification of the atherosclerosis degree for timely and appropriate prevention and treatment. Despite the existence of various risk factors and biomarkers, the efficacy of early identification and intervention of atherosclerosis has not been optimal [4]. This highlights the clinical need to explore specific and easily accessible biomarkers associated with atherosclerosis from a novel perspective, to improve risk stratification and facilitate early prevention and treatment.

The initiation and progression of atherosclerosis are associated with cell death, oxidative metabolism, inflammatory cell activation state, and mitochondrial dysfunction [5–8]. Mitochondrial dysfunction is commonly reflected by a reduction in mitochondrial deoxyribonucleic acid copy number (mtDNA-CN) and an overexpression of reactive oxygen species (ROS) in clinical research [9–16]. It has been reported that there is less mtDNA-CN and more ROS content in aortic and carotid atherosclerotic plaques compared to normal artery walls [17, 18]. However, assessing mitochondrial function in plaque is difficult in clinical settings because of the invasive nature of obtaining plaque by endarterectomy. Monocytes and lymphocytes, collectively referred to as peripheral blood mononuclear cells (PBMCs), are the major inflammatory cells involved in atherosclerosis [1], and they can be easily isolated from peripheral blood. A previous study suggested that mitochondrial dysfunction in peripheral blood inflammatory cells may correspond to that in atherosclerotic plaques [19]. However, there is still a lack of studies on the correlation between PBMC mitochondrial dysfunction and cervicocephalic atherosclerosis. Moreover, because of the systemic nature of atherosclerosis, local atherosclerotic plaque or single-artery stenosis does not accurately reflect the overall degree of atherosclerosis. Thus, further investigation is needed to explore the relationship between PBMC mitochondrial dysfunction and the overall degree of cervicocephalic atherosclerosis. In addition, previous studies have shown that lower mtDNA-CN is associated with a poor prognosis for stroke patients (including those with ischemic and hemorrhagic stroke) [15], but there are currently no related studies on mtDNA-CN or ROS content in PBMCs and functional outcomes in ischemic stroke patients, especially those with cervicocephalic atherosclerosis.

In previous studies, real-time quantitative polymerase chain reaction (qPCR) was used to detect mtDNA-CN [20–22]. Droplet digital polymerase chain reaction (ddPCR) is a new method developed in recent years that has the characteristics of absolute quantification and accurate analysis [23], and is superior to previous methods. In this study, ddPCR was used for the first time to measure mtDNA-CN in PBMCs and investigate its relationship with cervicocephalic atherosclerosis. ROS content is usually determined using a fluorescent probe. In addition, intracranial and cervical atherosclerotic burden (ICAB), as a new atherosclerosis assessment index, can quantify the degree and extent of cervicocephalic atherosclerosis as a whole. Our recent study showed that this indicator has a stronger vascular risk stratification value than regional atherosclerosis assessment [24]. In this study, mtDNA-CN, ROS content, ICAB and a modified Rankin Scale (mRS) were used to investigate the correlation between PBMC mitochondrial dysfunction and the overall degree of cervicocephalic atherosclerosis, along with the poor short-term functional prognosis of patients with AIS, in order to provide a new way for improving the early identification and risk stratification of cervicocephalic atherosclerosis in addition to the functional prognosis prediction of patients with AIS with cervicocephalic atherosclerosis.

## Materials and methods

### Study population

This single-center, prospective cohort study was performed in accordance with the Declaration of Helsinki, and was approved by the Ethics Committee of Xuanwu Hospital, Capital Medical University (Beijing, China) [approval number (2022)008, 26 January 2022]. All patients gave their informed consent before inclusion. Patients admitted to the cerebral vascular disease unit of the Department of Neurology at Xuanwu Hospital, Capital Medical University from 01 February 2022 to 30 April 2022 were consecutively enrolled.

The inclusion criteria were as follows: (1) at least 18 years old; (2) first-ever AIS confirmed by computed tomography or magnetic resonance imaging; (3) within 7 days after onset of symptoms; (4) undertook computed tomography angiography (CTA) successfully; and (5) AIS subtypes of large-artery atherosclerosis (LAA) and small-artery occlusion (SAO) with cervicocephalic atherosclerosis [25].

The exclusion criteria included: (1) a history of hemorrhagic stroke or subarachnoid hemorrhage; (2) symptomatic atherosclerotic coronary artery disease; (3) infection or chronic inflammatory disease (temperature ≥37.3°C, number of leukocytes >12 × 10^9^/L or <4 × 10^9^/L, use of anti-inflammatory drugs or antibiotics); (4) immunodeficiency or use of immunosuppressants; (5) mitochondrial myopathy or mitochondrial encephalomyopathy; (6) poor organ function; (7) hematological system diseases; and (8) malignant tumors.

### General characteristics

The patients’ age, sex, history of hypertension, diabetes mellitus, hyperlipidemia history, smoking history, and alcohol consumption were collected through interviews. Smoking was defined as having smoked within the last 12 months. Neurological deficit severity was estimated according to the National Institutes of Health Stroke Scale (NIHSS) upon admission [26]. Blood samples from all the enrolled patients were subjected to complete blood counts, biochemical tests, and coagulation function assays. Medical treatment during hospitalization was in accordance with AIS management and secondary prevention guidelines [27].

### Measurement of ROS content by fluorescence spectroscopy

In total, 10 mL of fasting venous blood was collected from each patient within 24 h after admission. PBMCs were isolated by density gradient centrifugation using Ficoll-Pauqe PLUS (17144002, Cytiva, Marlborough, MA, United States) within 2 hours of the blood sample being taken. The ROS content was then measured within 4 h by fluorescence spectroscopy. The PBMCs of each patient were resuspended in 500 μL of D-PBM buffer (201050, PBM, Tianjin, China) and then divided into a background tube and a stained tube. There were approximately 1 × 10^6^ PBMCs per tube. CellROX Green (C10444, Invitrogen, Carlsbad, CA, United States) was added to the stained tube to reach a final concentration of 5 μM. The two tubes were then incubated in a water bath at 37°C for 30 min, and the twice-washed PBMCs were placed in a 96-microwell plate (137101, Thermo Scientific, Waltham, MA, United States). The wells were defined as follows: negative well (100 μL D-PBS buffer), background well (100 μL unstained cell suspension), and stained well (100 μL stained cell suspension), respectively. Finally, a microplate reader (Varioskan Flash Multimode Reader, Thermo Scientific, Waltham, MA, United States) and SkanIt Software 2.4.5 were used to detect fluorescence intensity (FI) by fluorometry (excitation/emission: 485/520 nm), and the ROS content was then calculated using Equation 1:

$$\text{ROS content}=\left({\text{FI}}_{\text{stained}}{\;–\;\text{FI}}_{\text{background}}\right)/{\text{FI}}_{\text{negative}}$$ (1)

### Determination of mtDNA-CN by ddPCR

After isolating the PBMCs, an animal mtDNA column extraction kit (BTN80803, Beijing BioRab Technology, Beijing, China) was used to isolate the mtDNA. The mitochondrially encoded NADH dehydrogenase 1 (ND1) gene is located in the mitochondrial membrane, and the ribonuclease P/MRP 30 kDa subunit (RPP30) gene is a cellular housekeeping gene of the nucleus. ddPCR was used in separate reactions to measure the copy number of the mitochondrial ND1 gene (ND1-CN) and the nuclear RPP30 gene copy number (RPP30-CN). The mtDNA-CN quantification was calculated using Equation 2:

mtDNA‐CN=ND1‐CN/RPP30‐CN (2)

Amplification of the target DNA was performed in a 20 μL reaction mixture sample containing a ddPCR premixture (10 μL), mtDNA (1 μL), the restriction enzyme Hind III (0.3 μL), nuclease-free water (6.65 μL), a forward primer (0.9 μL), a reverse primer (0.9 μL), and a probe (0.25 μL). The sample was loaded into droplet generator (MicroDrop-100A, Forevergen, Guangzhou, Guangdong, China) to convert it into a water-in-oil droplet emulsion. The thermal cycling conditions consisted of a 10 min pre-denaturation at 95°C followed by 45 cycles of a 30 s denaturation at 95°C, a 1 min annealing-extension at 58°C for the ND1 reaction and at 61°C for the RPP30 reaction, a 10 min inactivation at 98°C, and a 4°C holding period. Finally, the amplified sample was analyzed using biochip analyzer (MicroDrop-100B, Forevergen, Guangzhou, Guangdong, China). The ND1-CN and RPP30-CN values were calculated using the QuantDrop system. The results were considered reliable when the number of droplets was >40,000.

The ND1 primers and probe used in this work were as follows:

forward primer, 5′-CCC​TAA​AAC​CCG​CCA​CAT​CT-3’;
reverse primer, 5′-GAG​CGA​TGG​TGA​GAG​CTA​AGG​T-3’;
probe, 5′-VIC/CCATCACCCTCTACATCACCGCCC/DBQ1-3’.

The following RPP30 primers and probe were considered:

forward primer, 5′-AGA​TTT​GGA​CCT​GCG​AGC​G-3’;
reverse primer, 5′-GAG​CGG​CTG​TCT​CCA​CAA​GT-3’;
probe, 5′-FAM/TTCTGACCTGAAGGCTCTGCGCG/BHQ1-3’.

### Quantification of the overall degree of atherosclerosis in cervicocephalic arteries using CTA

The CTA scan mode and the segments of cervicocephalic arteries described in our previous study were adopted in the present work [24]. Images of the cervicocephalic arteries were reconstructed and reviewed independently by two experienced radiologists who were unaware of the patients’ demographic and clinical information. Any discrepancies were discussed until a consensus was reached.

The degree of stenosis in the cervical arteries was evaluated based on the North American Symptomatic Carotid Endarterectomy Trial [28], and that in the intracranial arteries was evaluated based on the Warfarin-Aspirin Symptomatic Intracranial Disease Study [29]. The cervicocephalic arteries were divided into 19 segments as follows: 18 segments (9 bilateral arteries, including the subclavian, common carotid, extracranial carotid, extracranial vertebral, intracranial carotid, intracranial vertebral, posterior cerebral, middle cerebral and anterior cerebral arteries), plus one single segment (the basilar artery) [30]. We scored the most severe atherosclerotic lesion in each segment as follows: 0 points (no atherosclerotic lesion), 1 point (<50% stenosis or atherosclerotic plaque with no stenosis), 2 points (50%–69% stenosis), 3 points (70%–99% stenosis), and 4 points (occlusion), respectively [24]. ICAB was calculated by summing the points of each segment.

### Follow-up

All patients were followed up at 90 days after the onset of AIS via telephone interview with the patients or their long-term caregivers by an experienced physician who was blinded to the baseline data. The mRS was used to evaluate the post-stroke disability [31].

### Grouping of study subjects

ROS content, ICAB, and mtDNA-CN were divided into low, median, and high groups based on tertiles. In addition, ROS content was also classified as low or high according to the median. An unfavorable functional outcome was defined as an mRS >2, while a favorable functional outcome was defined as an mRS ≤2.

### Statistical analysis

The experimental data are presented as the mean ± standard deviation for normally distributed continuous variables, as the median (interquartile range) for non-normally distributed continuous variables, and as the count (%) for categorical variables. Continuous variables were compared using a Student’s t-test or a Mann-Whitney U test between two groups as appropriate, and using a one-way analysis of variance or a Kruskal-Wallis test with Bonferroni’s correction between three groups. Categorical variables were compared using a Chi-squared test or Fisher’s exact test.

Spearman’s correlation analysis was used to evaluate the correlation of mtDNA-CN and ROS content with ICAB, as well as that of mtDNA-CN with ROS content. For further exploration, the correlation of mtDNA-CN and ROS content with ICAB was assessed by multivariable linear regression analysis. The relationships between mitochondrial function (mtDNA-CN and ROS content) and ICAB were evaluated using Spearman’s correlation and multivariable linear regression. The relationship between mtDNA-CN and ROS content was assessed using Spearman’s correlation and multivariable logistic regression. Additionally, multivariable logistic regression was also used to examine the associations of mitochondrial function (mtDNA-CN and ROS content) and ICAB with 90-day unfavorable functional outcome (mRS >2). All tests were two-sided, and *p* < 0.05 was considered statistically significant. The statistical analyses were performed using SPSS Statistics (version 25.0; IBM, Armonk, NY, United States), and GraphPad Prism (version 8.0) was used for the preparation of figures.

## Results

**FIGURE 1:**
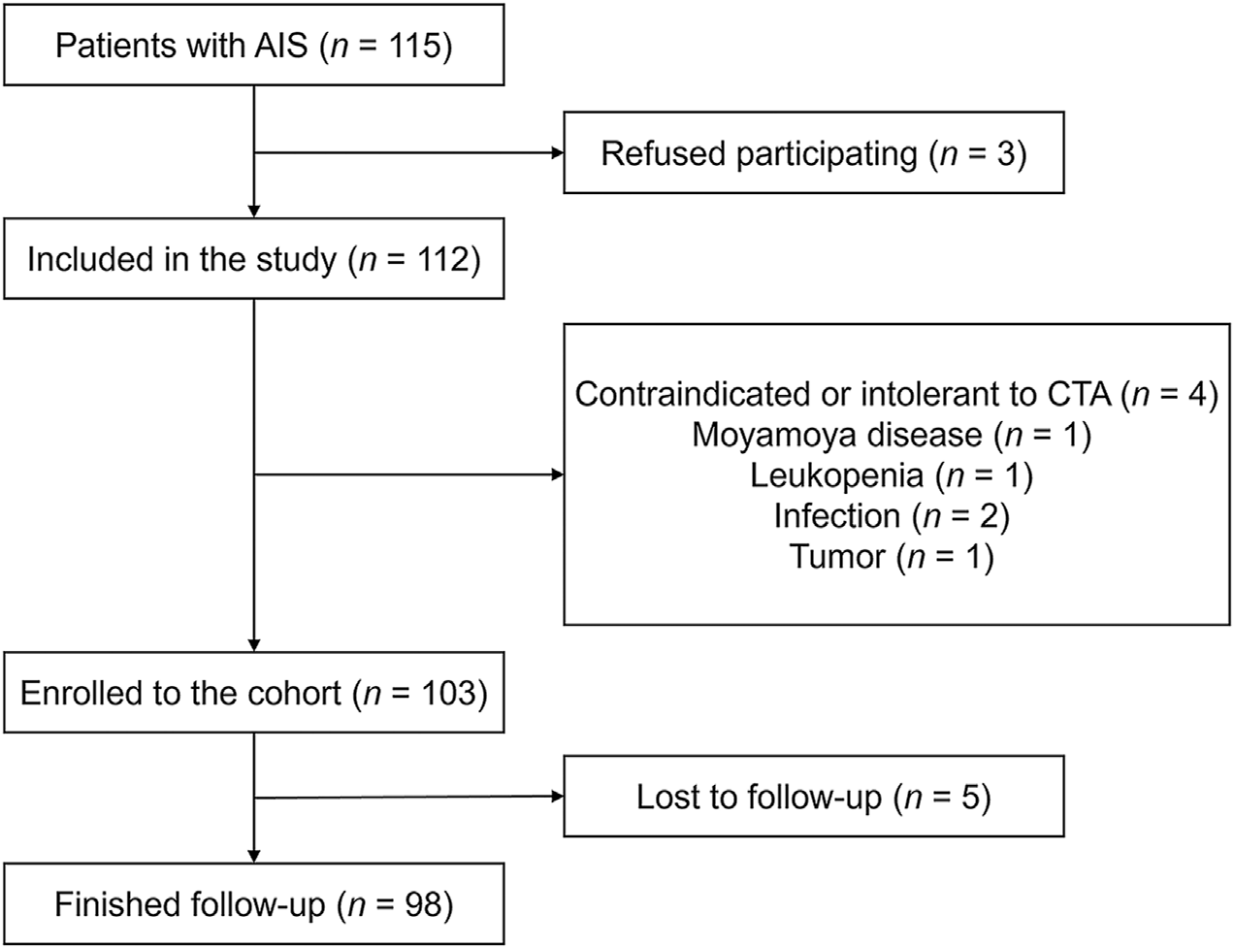
Flowchart of patients’ enrollment and follow-up. Abbreviations: AIS, acute ischemic stroke; CTA, computed tomography angiography.

Of the 115 participants with AIS without symptomatic atherosclerotic coronary artery disease, the following patients were excluded: those who refused to participate (*n* = 3), those with incomplete CTA (*n* = 4), those with Moyamoya disease (*n* = 1), those with leukopenia (*n* = 1), those with infection (*n* = 2), and those with a tumor (*n* = 1). Thus, there was a total of 103 patients enrolled in our cohort. Among them, 5 (4.9%) patients were lost to follow-up, and the study finished with 98 patients with complete information on mRS 90 days after ischemic stroke (Figure 1).

### Baseline characteristics and 90-day functional outcomes

Among the 98 patients who completed the 90-day follow-up, the mean age was 60.8 ± 11.5 years, and 67 patients (68.4%) were men. The median ICAB was 14 (range 7–20) points, and the median baseline mRS was 1 (range 0–3) point. The PBMC mitochondrial function was indicated by a median mtDNA-CN of 24.32 (range 13.27–37.97) ×10^2^ and a median ROS content of 5.71 (range 3.88–8.50) in the 98 patients, with no differences compared to patients lost to follow-up (*p* = 0.256, *p* = 0.914) (Supplementary Table S1). Patients in the high ICAB group (ICAB >18 points) had lower mtDNA-CN (*p* < 0.05) and higher ROS content (*p* < 0.05) than those in the low ICAB group (ICAB <10 points) (Table 1). At the end of the 90-day follow-up period, 24 patients (24.5%) had an unfavorable functional outcome (mRS >2) (Supplementary Table S2).

**TABLE 1:** Comparison of baseline characteristics of patients in low, median, and high ICAB groups.

Variables	Low ICAB (*n* = 34)	Median ICAB (*n* = 32)	High ICAB (*n* = 32)	*p* value
Demographics				
Age (years)	58.2 ± 13.5	64.8 ± 11.0	59.6 ± 8.58	0.050
Male patients (*n*)	20 (58.8)^a^	19 (59.4)^a^	28 (87.5)	0.018^b^
Clinical characteristics				
NIHSS (points)	1 (0–2)	1 (0–3)	3 (1–5)	0.028^b^
mRS (points)	1 (0–2)^a^	0 (0–3)^a^	3 (1–3)	0.013^b^
ICAB (points)	4 (1–7)^a^ ^,^ ^c^	15 (11–16)^a^	23 (20–25)	<0.001^b^
BMI (kg/m^2^)	26.01 ± 4.89	26.28 ± 3.61	26.05 ± 4.02	0.961
SBP (mmHg)	139.4 ± 22.1	138.2 ± 21.6	144.7 ± 18.6	0.421
DBP (mmHg)	87.8 ± 13.1	82.0 ± 11.1	89.0 ± 12.5	0.052
HbA1c (%)	5.60 (5.30–5.95)^a^	5.90 (5.40–7.48)	6.25 (5.50–7.88)	0.044^b^
FBG (mmol/L)	4.87 (4.24–5.36)^a^	5.26 (4.46–6.83)	5.92 (5.09–7.60)	0.001^b^
TC (mmol/L)	4.04 ± 1.05	4.21 ± 1.56	4.31 ± 1.26	0.363
TG (mmol/L)	1.23 (0.86–1.89)	1.42 (1.18–1.69)	1.52 (1.24–2.13)	0.067
LDL-C (mmol/L)	1.98 (1.76–2.96)	2.21 (1.42–2.90)	2.53 (1.69–3.35)	0.438
HDL-C (mmol/L)	1.17 ± 0.28^a^	1.12 ± 0.28^a^	0.96 ± 0.25	0.007^b^
Hcy (μmol/L)	16.94 (11.08–18.38)	16.29 (13.18–18.23)	14.95 (11.05–17.31)	0.394
UA (mmol/L)	316.41 ± 96.86	300.47 ± 87.54	322.66 ± 88.93	0.606
hs-CRP (mg/L)	1.50 (0.68–4.38)	1.37 (0.57–5.07)	3.68 (0.75–5.13)	0.462
FIB (g/L)	3.02 ± 0.78	3.12 ± 1.02	3.54 ± 0.98	0.690
D-Dimer (mmol/L)	0.26 (0.20–0.49)	0.26 (0.21–0.36)	0.25 (0.14–0.34)	0.547
Risk factors				
History of hypertension (*n*)	20 (58.8)	23 (71.9)	26 (81.3)	0.133
History of diabetes (*n*)	4 (11.8)	10 (31.3)	11 (34.4)	0.072
History of hyperlipidemia (*n*)	15 (44.1)	12 (37.5)	15 (46.9)	0.738
Smoking history (*n*)	10 (29.4)	11 (34.4)	18 (56.3)	0.063
Alcohol consumption (*n*)	8 (23.5)	11 (34.4)	16 (50.0)	0.079
PBMC characteristics				
PBMC count (×10^9^/L)	2.29 ± 0.96	2.48 ± 0.66	2.37 ± 0.98	0.334
mtDNA-CN (×10^2^)	32.61 (23.33–50.94)^a^	28.28 (14.28–40.89)^a^	12.61 (6.02–21.65)	<0.001^b^
ROS content	4.09 (2.75–5.10)^a^ ^,^ ^c^	7.16 (4.94–8.14)	8.75 (5.06–12.18)	<0.001^b^

Data presented as mean ± standard deviation, median (interquartile range), or *n* (%). ICAB was grouped by tertile: low ICAB <10 points, median ICAB = 10 ∼ 18 points, and high ICAB >18 points.

Abbreviations: ICAB, intracranial and cervical atherosclerotic burden; NIHSS, national institute of health stroke scale; mRS, modified Rankin Scale; BMI, body mass index; SBP, systolic blood pressure; DBP, diastolic blood pressure; HbA1c, glycated hemoglobin; FBG, fasting blood glucose; TC, total cholesterol; TG, triglyceride; LDL-C, low-density lipoprotein cholesterol; HDL-C, high-density lipoprotein cholesterol; Hcy, homocysteine; UA, serum uric acid; hs-CRP, hypersensitive C-reactive protein; FIB, fibrinogen; PBMC, peripheral blood mononuclear cells; mtDNA-CN, mitochondrial deoxyribonucleic acid copy number; ROS, reactive oxygen species.

^a^ *p* < 0.05 compared with the high ICAB group.

^b^ Statistically significant differences (*p* value < 0.05).

^c^ *p* < 0.05 compared with the median ICAB group.

The kappa value for inter-rater reliability in ICAB was 0.929 (*p* < 0.001).

### Relationship between PBMC mitochondrial dysfunction and ICAB

**FIGURE 2:**
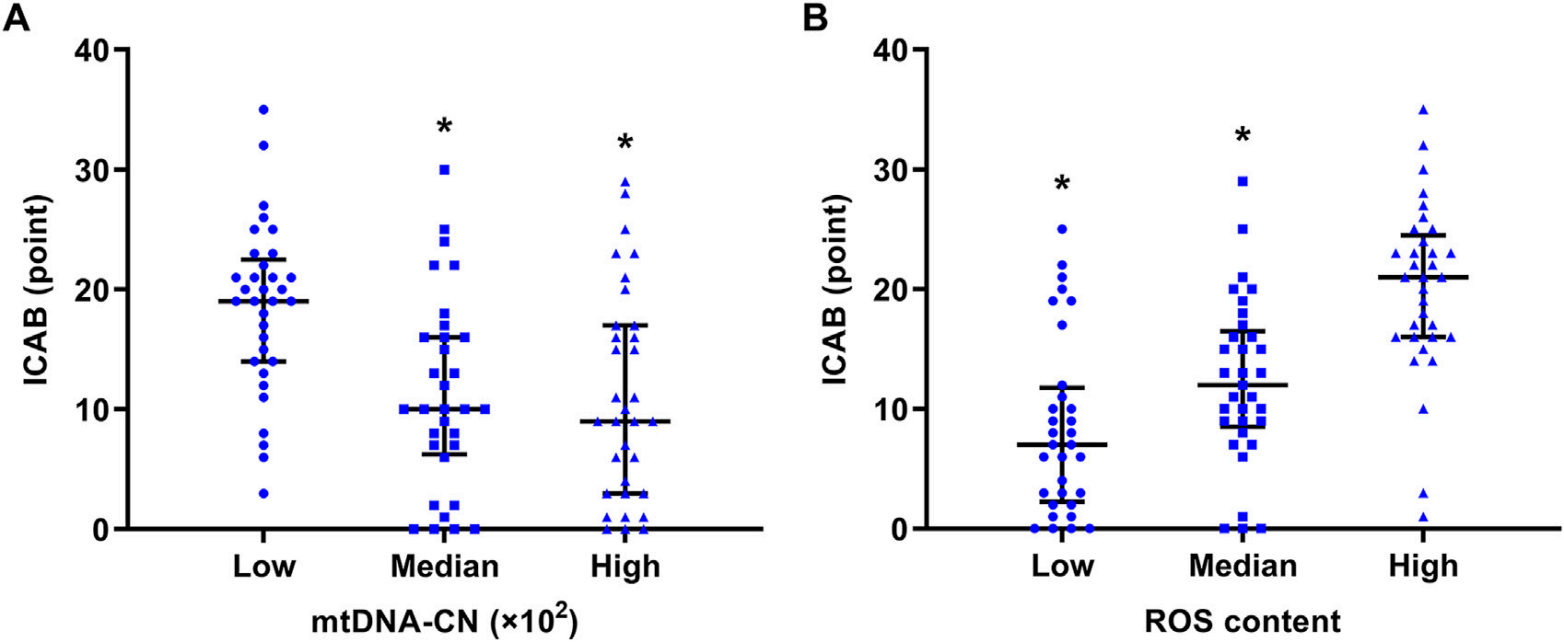
Comparison of ICAB between different groups of mtDNA-CN and ROS content. **(A)** ICAB of the low mtDNA-CN group was significantly higher than that of the median and high mtDNA-CN groups. The mtDNA-CN was grouped by tertile: low mtDNA-CN < 18.08 ×10^2^, median mtDNA-CN = (18.08 ∼ 32.83) ×10^2^, high mtDNA-CN >32.83 ×10^2^. ^*^ *p* < 0.001 vs the low mtDNA-CN group. **(B)** ICAB of the high ROS content group was significantly higher than that of the low and median ROS content groups. ROS content was grouped by tertile: low ROS content <4.64, median ROS content = 4.64 ∼ 7.85, high ROS content >7.85. ^*^ *p* < 0.001 vs. the high ROS content group. ICAB is represented as the median (interquartile range). Abbreviations: ICAB, intracranial and cervical atherosclerotic burden; mtDNA-CN, mitochondrial deoxyribonucleic acid copy number; ROS, reactive oxygen species.

**FIGURE 3:**
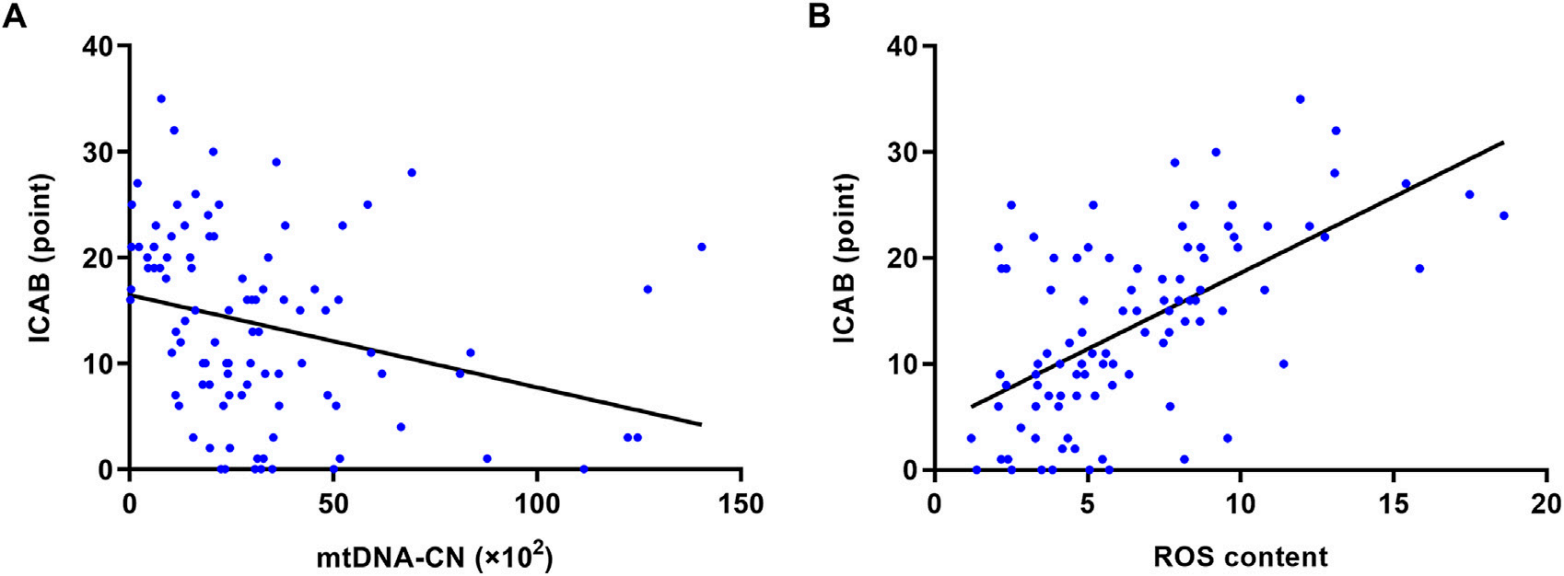
Spearman’s correlation analysis of mtDNA-CN, ROS content and ICAB. **(A)** Correlation between mtDNA-CN and ICAB (r = −0.39, *p* < 0.001). **(B)** Correlation between ROS content and ICAB (r = 0.58, *p* < 0.001). Abbreviations: ICAB, intracranial and cervical atherosclerotic burden; mtDNA-CN, mitochondrial deoxyribonucleic acid copy number; ROS, reactive oxygen species.

It is clear from Figure 2 that ICAB was found to be higher in each group with severe PBMC mitochondrial dysfunction (low mtDNA-CN <18.08 ×10^2^, high ROS content >7.85). Spearman’s correlation analysis showed a negative correlation between mtDNA-CN and ICAB (r = −0.39, *p* < 0.001) (Figure 3A), and a positive correlation between ROS content and ICAB (r = 0.58, *p* < 0.001) (Figure 3B). After adjusting for age, sex, PBMC count, and the parameters with *p* < 0.1 in univariable analysis (Supplementary Table S3), mtDNA-CN was found to be independently and negatively associated with ICAB (adjusted β = −0.099, 95%CI = −0.153 ∼ −0.044, *p* < 0.001), and ROS content was also positively associated independently with ICAB (adjusted β = 1.275, 95%CI = 0.885–1.665, *p* < 0.001) in multivariable linear regression analysis (Table 2).

**TABLE 2:** Multivariable linear regression of mtDNA-CN, ROS content and ICAB.

PBMC mitochondrial function	ICAB		
	β (95%CI)^a^	*p* value	R^2^
mtDNA-CN	−0.099 (−0.153 ∼ −0.044)	<0.001^b^	0.413
ROS content	1.275 (0.885–1.665)	<0.001^b^	0.549

^a^ Adjusted for age, sex, history of hypertension, history of diabetes, alcohol consumption, glycated hemoglobin, fasting blood glucose, triglyceride, high-density lipoprotein cholesterol, fibrinogen, and PBMC count.

Abbreviations: ICAB, intracranial and cervical atherosclerotic burden; mtDNA-CN, mitochondrial deoxyribonucleic acid copy number; ROS, reactive oxygen species; CI, confidence interval.

^b^ Statistically significant differences (*p* value < 0.05).

### Relationship between mtDNA-CN and ROS content

**FIGURE 4:**
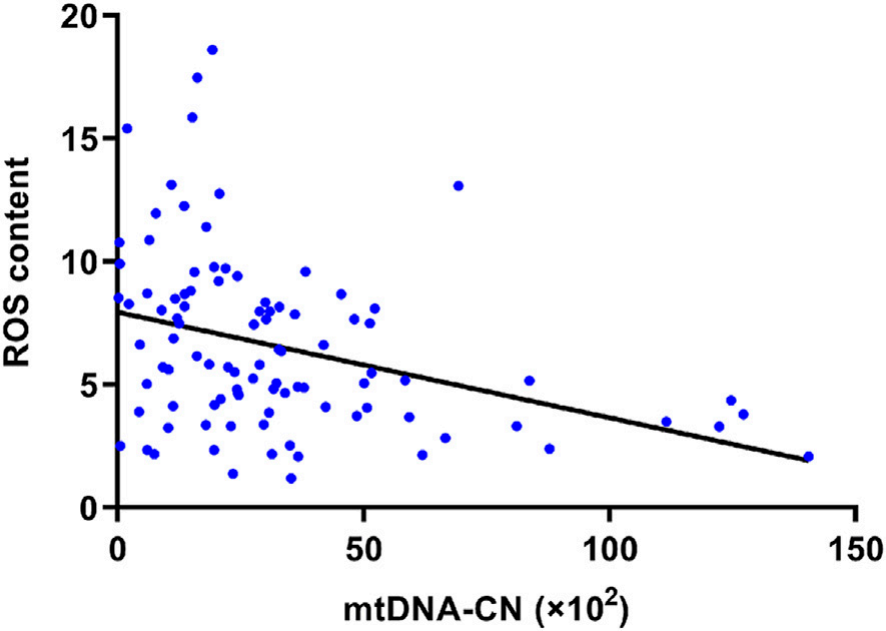
Spearman’s correlation analysis of mtDNA-CN and ROS content. The ROS content of PBMCs was negatively related to its mtDNA-CN (r = −0.38, *p* < 0.001). Abbreviations: mtDNA-CN, mitochondrial deoxyribonucleic acid copy number; ROS, reactive oxygen species.

Figure 4 shows the significant negative correlation between mtDNA-CN and ROS content in PBMCs in a Spearman’s correlation analysis (r = −0.38, *p* < 0.001). After adjusting for age, sex, diabetes history, and alcohol consumption in multivariable logistic regression, the risk of high ROS content decreased with elevated mtDNA-CN (adjusted OR = 0.959, 95%CI = 0.933–0.986, *p* = 0.003) (Table 3; Supplementary Table S4).

**TABLE 3:** Logistic regression analysis of mtDNA-CN and high ROS content.

Variable	High ROS content			
	Univariable analysis		Multivariable analysis	
	OR (95%CI)	*p* value	Adjusted OR (95%CI)^a^	*p* value
mtDNA-CN (per 1 × 10^2^ increased)	0.961 (0.939–0.984)	0.001^b^	0.959 (0.933–0.986)	0.003^b^

^a^ Adjusted for age, sex, history of diabetes, and alcohol consumption.

ROS content was grouped by the median, with high ROS content ≥5.71.

Abbreviations: mtDNA-CN, mitochondrial deoxyribonucleic acid copy number; ROS, reactive oxygen species; OR, odds ratio; CI, confidence interval.

^b^ Statistically significant differences (*p* value < 0.05).

### Relationship between ICAB and a 90-day functional outcome of AIS

Compared to patients with a favorable functional outcome, those with an unfavorable functional outcome (90-day mRS >2) had higher levels of low-density lipoprotein cholesterol (*p* = 0.033), NIHSS (*p* < 0.001), ICAB (*p* < 0.001), and ROS content (*p* = 0.010), but lower levels of mtDNA-CN (*p* = 0.001) (Supplementary Table S2). Multivariable logistic regression analysis revealed that ICAB was independently related to a 90-day unfavorable functional outcome after adjusting for age, sex, PBMC count, total cholesterol, low-density lipoprotein cholesterol, and NIHSS (adjusted OR = 1.127, 95%CI = 1.021–1.244, *p* = 0.018) (Table 4; Supplementary Table S5).

**TABLE 4:** Logistic regression analysis of ICAB, mtDNA-CN, ROS content, and 90-day unfavorable functional outcome.

Variables	90-day unfavorable functional outcome			
	Univariable analysis		Multivariable analysis	
	Or (95%CI)	*p* value	Adjusted OR (95%CI)^a^	*p* value
ICAB (per point increased)	1.115 (1.045 ∼ 1.190)	0.001^b^	1.127 (1.021 ∼ 1.244)	0.018^b^
mtDNA-CN (per 1 × 10^2^ increased)	0.953 (0.921 ∼ 0.987)	0.006^b^	0.911 (0.850 ∼ 0.976)	0.008^b^
ROS content (per level increased)	1.265 (1.097 ∼ 1.459)	0.001^b^	1.523 (1.172 ∼ 1.981)	0.002^b^

^a^ Adjusted for age, sex, PBMC, count, total cholesterol, low-density lipoprotein cholesterol, and NIHSS.

A 90-day unfavorable functional outcome was defined as mRS >2 points.

Abbreviations: ICAB, intracranial and cervical atherosclerotic burden; mtDNA-CN, mitochondrial deoxyribonucleic acid copy number; ROS, reactive oxygen species; OR, odds ratio; CI, confidence interval.

^b^ Statistically significant differences (*p* value < 0.05).

### Relationship between PBMC mitochondrial dysfunction and a 90-day functional outcome of AIS

Multivariable logistic regression analysis showed that after adjusting for age, sex, PBMC count, total cholesterol, low-density lipoprotein cholesterol, and NIHSS, patients with higher mtDNA-CN had a decreased risk of a 90-day unfavorable functional outcome (adjusted OR = 0.911, 95%CI = 0.850–0.976, *p* = 0.008), while patients with higher ROS content had an increased risk of 90-day unfavorable functional outcome (adjusted OR = 1.523, 95%CI = 1.172 ∼ 1.981, *p* = 0.002) (Table 4; Supplementary Table S5).

## Discussion

In this study, we found that mtDNA-CN and ROS content of PBMCs were negatively and positively correlated with ICAB, respectively. With the increase of the mtDNA-CN level, the ROS content and the risk of mRS >2 decreased significantly. To our knowledge, this is the first study to suggest that severe PBMC mitochondrial dysfunction is associated with extensive and severe cervicocephalic atherosclerosis, in addition to increased risk of short-term unfavorable functional outcomes in patients with AIS.

Mitochondrial dysfunction may be involved in atherogenesis and has been found in local carotid plaque [18, 32]. Another study of 11 participants revealed decreased mtDNA-CN in coronary artery plaques and a reduction in peripheral blood leukocytes [19], indicating that mitochondrial dysfunction in peripheral blood inflammatory cells could correspond to that in atherosclerotic plaques. mtDNA damage in PBMC has also been reported to be more significant in patients with atherosclerotic cardiovascular disease than in controls without atherosclerosis [33]. Similarly, our study confirmed that the mtDNA-CN of PBMCs was closely related to cervicocephalic atherosclerosis. Thus, it is possible to speculate that similar to the mitochondrial dysfunction observed in plaques, mitochondrial dysfunction in peripheral blood inflammatory cells may also correlate with atherosclerosis. It is also worth noting that mice with mtDNA damage or greater oxidative stress in peripheral blood inflammatory cells were reported to have larger aortic atherosclerotic plaques than those without [32, 34], showing the aggravating role of mitochondrial dysfunction in the progression of local atherosclerosis. However local atherosclerosis may not accurately reflect the overall degree of atherosclerosis. The impact of mitochondrial dysfunction on overall atherosclerosis needs to be further explored. The relationship between mtDNA-CN in peripheral blood cells and the total degree of atherosclerosis has only been observed in coronary arteries, with inconsistent results and without ROS content assessment [20, 21]. Furthermore, compared to coronary arteries and aortic arteries, cervicocephalic arteries provide a wider range of blood supply with a longer arterial course. Thus, the association, especially between mitochondrial dysfunction in peripheral blood inflammatory cells and the overall degree of cervicocephalic atherosclerosis (ICAB) warrants further investigation. Our findings demonstrated that patients with AIS with severe PBMC mitochondrial dysfunction (lower mtDNA-CN and higher ROS content) are at a higher risk for an extensive and severe degree of overall cervicocephalic atherosclerosis (higher ICAB) (Table 2). Therefore, assessing PBMC mitochondrial dysfunction has potential value in risk stratification for cervicocephalic atherosclerosis in clinical work.

The mitochondrial dysfunction of PBMCs in patients with AIS was evaluated in our study from the perspectives of gene expression and oxidative metabolism, as indicated by mtDNA-CN and ROS content. We found that ROS content gradually increased as mtDNA-CN in PBMCs decreased (Figure 4), suggesting that mtDNA damage and oxidative metabolic injury may not be independent of each other in the presence of atherosclerosis. It should be emphasized that mtDNA lacks histone protection and effective mtDNA repair, and is close to the electron transport chain where ROS are generated [35]. As a result, mtDNA is easy to be damaged by ROS and oxidative stress. Increased ROS content in inflammatory cells may also lead to a decrease in mtDNA-CN, which in turn may lead to a decline in respiratory chain function, an imbalance in cellular oxidative metabolism, and the promotion of further ROS production, thus forming a vicious cycle [36]. Therefore, the decrease in mtDNA-CN and the increase in ROS content in PBMCs are likely to promote each other and may aggravate atherosclerosis. This finding provides some clues for further exploration of the possible mechanisms underlying PBMC mitochondrial dysfunction in patients with AIS with cervicocephalic atherosclerosis.

Previous studies have shown that lower mtDNA-CN in peripheral blood leukocytes can predict poorer outcomes in stroke patients [15]. We conducted this study especially with patients with AIS with LAA and SAO subtypes, so as to better observe the relationship between mitochondrial dysfunction and the prognosis of patients with AIS with cervicocephalic atherosclerosis. Our results showed that both the decrease of mtDNA-CN and the increase of ROS content in PBMCs were independently associated with a 90-day unfavorable functional outcome after AIS (Table 4). Except for the poor outcomes caused by severe atherosclerosis in the cervicocephalic arteries [2, 3, 24], the association between PBMC mitochondrial dysfunction and the functional outcomes of AIS may also involve other possible mechanisms unrelated to atherosclerosis. The mtDNA-CN of peripheral blood leukocytes and other types of cells, such as neurons and endothelial cells may exhibit similar trends of change, and adequate mtDNA-CN in these cells could enhance the repair ability of neurons and the blood-brain barrier after stroke [15]. In addition, low mtDNA-CN may increase the proportion of pro-inflammatory macrophages [9], resulting in the release of more inflammatory factors that exacerbate neuronal damage [37]. Moreover, the increased ROS content could promote endothelial cell injury and apoptosis, in addition to glucose metabolism dysfunction [7, 38–40]. These cellular and tissue damages might affect the functional recovery of patients with AIS, and these potential mechanisms need further exploration.

Finally, it must be mentioned that our study has certain limitations. (i) As a single-center study with a relatively small sample size, the results of this study should be generalized to other populations with caution. (ii) The majority of patients with AIS in this study exhibited mild neurological deficits, and further research should include patients with different degrees of neurological impairment for a more comprehensive analysis. (iii) While PBMC mitochondrial function and the majority of the clinical characteristics did not differ between patients with and without a completed follow-up, the lower ICAB scores observed in the latter group may indicate the potential bias. Further studies with a larger sample size are needed to reduce this bias. (iv) In addition to functional outcomes, we will continue to follow up on further cardio-cerebral vascular events in patients with AIS to comprehensively investigate the correlation between PBMC mitochondrial dysfunction and poor AIS outcomes. (v) To explore the mechanism by which PBMC mitochondrial dysfunction drives the development of the degree of atherosclerosis, interventional experiments such as oxidative stress inhibition assays should also be conducted.

## Conclusion

In summary, this study demonstrated that PBMC mitochondrial dysfunction may play an important role in indicating the extensive and severe overall cervicocephalic atherosclerotic burden and a poor short-term functional outcome of patients with AIS. Given the advantages of the convenient and non-invasive approach to measuring PBMC mitochondrial dysfunction, this study may provide a novel and feasible way to optimize the early identification and risk stratification of cervicocephalic atherosclerosis and functional prognosis prediction of patients with AIS with cervicocephalic atherosclerosis. Further investigation is needed to understand the complex mechanisms between mitochondrial dysfunction in peripheral blood inflammatory cells and atherosclerosis

## Data Availability

The original contributions presented in the study are included in the article/Supplementary Material, further inquiries can be directed to the corresponding authors.
