# Supplementary material for: Dependence of mitochondrial dysfunction in peripheral blood mononuclear cells on cervicocephalic atherosclerotic burden in acute ischemic stroke
Source: Exp Biol Med (Maywood). 2025 Jul 1;250:10624. doi: 10.3389/ebm.2025.10624 (PMC12259479; doi:10.3389/ebm.2025.10624)
Supplement: Supplementary file 1 [file Table1.docx]

**TABLE S1** Comparison of baseline characteristics of patients with and without 90-day follow-up.

| Variables | 90-day follow-up  (*n* = 98) | Loss to follow-up  (*n* = 5) | *p* value |
| --- | --- | --- | --- |
| Demographics |  |  |  |
| Age (years) | 60.8 ± 11.5 | 60.0 ± 19.6 | 0.883 |
| Male (*n*) | 67 (68.4) | 5 (100) | 0.315 |
| Clinical characteristics |  |  |  |
| NIHSS (point) | 1 (0-4) | 0 (0-0) | 0.012^*^ |
| mRS (point) | 1 (0-3) | 1 (1-1) | 1.000 |
| ICAB (point) | 14 (7-20) | 0 (0-3) | 0.001^*^ |
| BMI (kg/m^2^) | 26.11 ± 4.18 | 26.42 ± 4.18 | 0.875 |
| SBP (mmHg) | 140.7 ± 20.8 | 131.0 ± 24.4 | 0.313 |
| DBP (mmHg) | 86.0 ± 12.5 | 83.6 ± 13.6 | 0.640 |
| HbA1c (%) | 5.80 (5.40-6.93) | 6.60 (5.10-8.80) | 0.628 |
| FBG (mmol/L) | 5.25 (4.60-6.88) | 6.21 (4.97-8.62) | 0.319 |
| TC (mmol/L) | 4.18 ± 1.29 | 4.10 ± 0.57 | 0.884 |
| TG (mmol/L) | 1.40 (1.04-1.87) | 1.49 (1.18-2.05) | 0.645 |
| LDL-C (mmol/L) | 2.07 (1.62-3.01) | 2.07 (0.39-2.48) | 0.994 |
| HDL-C (mmol/L) | 1.09 ± 0.28 | 1.02 ± 0.30 | 0.605 |
| Hcys (μmol/L) | 16.38 (11.38-17.42) | 17.00 (10.35-17.29) | 0.662 |
| UA (mmol/L) | 313.25 ± 90.86 | 319.40 ± 40.83 | 0.881 |

**TABLE S1** (*Continued*) Comparison of baseline characteristics of patients with and without 90-day follow-up.

| Variables | 90-day follow-up  (*n* = 98) | Loss to follow-up  (*n* = 5) | *p* value |
| --- | --- | --- | --- |
| hs-CRP (mg/L) | 1.76 (0.66-4.56) | 0.93 (0.57-7.78) | 0.896 |
| FIB (g/L) | 3.22 ± 0.95 | 2.98 ± 0.66 | 0.574 |
| D-Dimer (mmol/L) | 0.25 (0.20-0.37) | 0.35 (0.18-0.73) | 0.593 |
| Risk factors |  |  |  |
| Hypertension history (*n*) | 69 (70.4) | 4 (80.0) | 1.000 |
| Diabetes history (*n*) | 25 (25.5) | 2 (40.0) | 0.844 |
| Hyperlipidemia history (*n*) | 42 (4.9) | 3 (60.0) | 0.771 |
| Smoking history (*n*) | 39 (39.8) | 3 (60.0) | 0.667 |
| Alcohol consumption (*n*) | 35 (35.7) | 3 (60.0) | 0.533 |
| PBMC characteristics |  |  |  |
| PBMC count (×10^9^/L) | 2.38 ± 0.87 | 2.20 ± 0.56 | 0.012^*^ |
| mtDNA-CN (×10^2^) | 24.32 (13.27-37.97) | 30.93 (13.46-36.22) | 0.256 |
| ROS content | 5.71 (3.88-8.50) | 4.14 (1.85-7.72) | 0.914 |

Data presented as mean ± standard deviation, median (interquartile range) or *n* (%).

Abbreviations: ICAB, intracranial and cervical atherosclerotic burden; NIHSS, National Institute of Health Stroke Scale; mRS, modified Rankin Scale; BMI, body mass index; SBP, systolic blood pressure; DBP, diastolic blood pressure; HbA1c, glycated hemoglobin; FBG, fasting blood glucose; TC, total cholesterol; TG, triglyceride; LDL-C, low-density lipoprotein cholesterol; HDL-C, high-density lipoprotein cholesterol; Hcys, homocysteine; UA, serum uric acid; hs-CRP, hypersensitive C-reactive protein; FIB, fibrinogen; PBMC, peripheral blood mononuclear cells; mtDNA-CN, mitochondrial deoxyribonucleic acid copy number; ROS, reactive oxygen species.

^*^Statistically significant differences (*p* value <0.05).

**TABLE S2** Comparison of baseline characteristics of patients in different 90-day mRS groups.

| Variables | 90-day mRS ≤2  (*n* = 74) | 90-day mRS >2  （*n* = 24） | *p* value |
| --- | --- | --- | --- |
| Demographics |  |  |  |
| Age (years) | 60.8 ± 12.2 | 60.8 ± 9.5 | 0.994 |
| Male (*n*) | 49 (66.2) | 18 (75.0) | 0.421 |
| Clinical characteristics |  |  |  |
| NIHSS (point) | 1 (0-2) | 5 (3-9) | <0.001^*^ |
| ICAB (point) | 12 ± 8 | 19 ± 9 | <0.001^*^ |
| BMI (kg/m^2^) | 25.74 ± 4.08 | 27.27 ± 4.36 | 0.118 |
| SBP (mmHg) | 139.2 ± 21.0 | 145.4 ± 19.8 | 0.210 |
| DBP (mmHg) | 86.0 ± 12.6 | 87.4 ± 12.5 | 0.633 |
| HbA1c (%) | 5.75 (5.40-6.75) | 5.90 (5.43-7.45) | 0.588 |
| FBG (mmol/L) | 5.23 (4.49-6.59) | 5.35 (4.77-7.37) | 0.173 |
| TC (mmol/L) | 3.76 (3.25-4.47) | 4.61 (3.22-5.70) | 0.072 |
| TG (mmol/L) | 1.42 (1.07-1.85) | 1.36 (0.96-2.05) | 0.670 |
| LDL-C (mmol/L) | 2.01 (1.58-2.76) | 3.00 (1.75-3.45) | 0.033^*^ |
| HDL-C (mmol/L) | 1.11 ± 0.27 | 1.01 ± 0.30 | 0.111 |
| Hcys (μmol/L) | 16.58 (11.48-17.41) | 14.75 (10.85-18.33) | 0.898 |
| UA (mmol/L) | 309.20 ± 95.77 | 325.71 ± 74.09 | 0.442 |
| hs-CRP (mg/L) | 1.50 (0.58-4.50) | 3.74 (0.75-4.80) | 0.326 |

**TABLE S2** (*Continued*) Comparison of baseline characteristics of patients in different 90-day mRS groups.

| Variables | 90-day mRS ≤2  (*n* = 74) | 90-day mRS >2  （*n* = 24） | *p* value |
| --- | --- | --- | --- |
| FIB (mmol/L) | 3.05 (2.50-3.77) | 3.20 (2.60-3.91) | 0.527 |
| D-Dimer (mmol/L) | 0.25 (0.20-0.34) | 0.27 (0.13-0.61) | 0.875 |
| Risk factors |  |  |  |
| Hypertension history *(n*) | 50 (67.6) | 19 (79.2) | 0.279 |
| Diabetes history (*n*) | 20 (27.0) | 5 (20.8) | 0.545 |
| Hyperlipidemia history (*n*) | 33 (44.6) | 9 (37.5) | 0.542 |
| Smoking (*n*) | 29 (39.2) | 10 (41.7) | 0.829 |
| Alcohol consumption (*n*) | 26 (25.1) | 9 (37.5) | 0.834 |
| PBMC characteristics |  |  |  |
| PBMC count (×10^9^/L) | 2.34 (1.78-2.72) | 2.38 (1.90-2.91) | 0.716 |
| mtDNA-CN (×10^2^) | 30.14 (17.48-48.25) | 15.06 (9.47-20.83) | 0.001^*^ |
| ROS content | 5.36 (3.78-7.97) | 9.00 (4.19-12.18) | 0.010^*^ |

Data presented as mean ± standard deviation, median (interquartile range) or *n* (%).

Abbreviations: mRS, modified Rankin Scale; NIHSS, National Institute of Health Stroke Scale; ICAB, intracranial and cervical atherosclerotic burden; BMI, body mass index; SBP, systolic blood pressure; DBP, diastolic blood pressure; HbA1c, glycated hemoglobin; FGB, fasting blood glucose; TC, total cholesterol; TG, triglyceride; LDL-C, low-density lipoprotein cholesterol; HDL-C, high-density lipoprotein cholesterol; Hcys, homocysteine; UA, serum uric acid; hs-CRP, hypersensitive C-reactive protein; FIB, fibrinogen; PBMC, peripheral blood mononuclear cells; mtDNA-CN, mitochondrial deoxyribonucleic acid copy number; ROS, reactive oxygen species.

^*^Statistically significant differences (*p* value <0.05).

**TABLE S3** Univariable linear regression of clinical variables and ICAB.

| Variables | ICAB | |
| --- | --- | --- |
|  | β (95%CI) | *p* value |
| Age (years) | 0.062 (-0.088 ~ 0.213) | 0.413 |
| Male | 4.084 (0.452 ~ 7.717) | 0.028^*^ |
| BMI (kg/m^2^) | 0.102 (-0.314 ~ 0.518) | 0.628 |
| SBP (mmHg) | 0.048 (-0.035 ~ 0.131) | 0.257 |
| DBP (mmHg) | 0.033 (-0.106 ~ 0.172) | 0.641 |
| HbA1c (%) | 1.670 (0.590 ~ 2.749) | 0.003^*^ |
| FBG (mmol/L) | 1.292 (0.602 ~ 1.982) | <0.001^*^ |
| TC (mmol/L) | 0.633 (-0.706 ~ 1.972) | 0.350 |
| TG (mmol/L) | 3.355 (1.354 ~ 5.356) | 0.001^*^ |
| LDL-C (mmol/L) | 0.717 (-0.933 ~ 2.367) | 0.391 |
| HDL-C (mmol/L) | -8.637 (-14.528 ~ -2.746) | 0.004^*^ |
| Hcys (μmol/L) | -0.059 (-0.210 ~ 0.091) | 0.437 |
| UA (mmol/L) | -0.002 (-0.021 ~ 0.018) | 0.869 |
| hs-CRP (mg/L) | 0.180 (-0.127 ~ 0.487) | 0.247 |
| FIB (g/L) | 2.364 (0.570 ~ 4.157) | 0.010^*^ |
| D-Dimer (mmol/L) | -0.219 (-1.045 ~ 0.607) | 0.600 |
| Hypertension history | 4.517 (0.833 ~ 8.201) | 0.017^*^ |
| Diabetes history | 5.231 (1.400 ~ 9.062) | 0.008^*^ |
| Hyperlipidemia history | -0.036 (-3.537 ~ 3.465) | 0.984 |

**TABLE S3** (*Continued*) Univariable linear regression of clinical variables and ICAB.

| Variables | ICAB | |
| --- | --- | --- |
|  | β (95%CI) | *p* value |
| Smoking history | 2.731 (-0.765 ~ 6.228) | 0.124 |
| Alcohol consumption | 4.845 (1.35 ~ 8.32) | 0.007^*^ |
| PBMC count (×10^9^/L) | -0.459 (-2.451 ~ 1.534) | 0.649 |
| mtDNA-CN (×10^2^) | -0.087 (-0.145 ~ -0.029) | 0.004^*^ |
| ROS content | 1.435 (1.044 ~ 1.827) | <0.001^*^ |

Abbreviations: ICAB, intracranial and cervical atherosclerotic burden; BMI, body mass index; SBP, systolic blood pressure; DBP, diastolic blood pressure; HbA1c, glycated hemoglobin; FBG, fasting blood glucose; TC, total cholesterol; TG, triglyceride; LDL-C, low-density lipoprotein cholesterol; HDL-C, high-density lipoprotein cholesterol; Hcys, homocysteine; UA, serum uric acid; hs-CRP, hypersensitive C-reactive protein; FIB, fibrinogen; PBMC, peripheral blood mononuclear cells; mtDNA-CN, mitochondrial deoxyribonucleic acid copy number; ROS, reactive oxygen species; CI, confidence intervals.

^*^Statistically significant differences (*p* value <0.05).

**TABLE S4** Univariable logistic regression of clinical variables and high ROS content.

| Variables | High ROS content | |
| --- | --- | --- |
|  | OR (95%CI) | *p* value |
| Age (years) | 1.034 (0.997 ~ 1.072) | 0.069 |
| Male | 2.075 (0.870 ~ 4.949) | 0.100 |
| BMI (kg/m^2^) | 0.996 (0.906 ~ 1.096) | 0.942 |
| SBP (mmHg) | 1.008 (0.988 ~ 1.028) | 0.435 |
| DBP (mmHg) | 1.003 (0.972 ~ 1.036) | 0.837 |
| HbA1c (%) | 1.196 (0.910 ~ 1.572) | 0.200 |
| FBG (mmol/L) | 1.142 (0.951 ~ 1.371) | 0.155 |
| TC (mmol/L) | 0.905 (0.664 ~ 1.234) | 0.528 |
| TG (mmol/L) | 1.099 (0.674 ~ 1.793) | 0.704 |
| LDL-C (mmol/L) | 0.872 (0.595 ~ 1.279) | 0.484 |
| HDL-C (mmol/L) | 0.543 (0.132 ~ 2.239) | 0.398 |
| Hcys (μmol/L) | 1.013 (0.977 ~ 1.051) | 0.481 |
| UA (mmol/L) | 1.000 (0.996 ~ 1.004) | 0.966 |
| hs-CRP (mg/L) | 0.977 (0.909 ~ 1.050) | 0.532 |
| FIB (g/L) | 1.091 (0.714 ~ 1.667) | 0.688 |
| D-Dimer (mmol/L) | 0.559 (0.222 ~ 1.409) | 0.218 |
| Hypertension history | 0.961 (0.403 ~ 2.288) | 0.928 |
| Diabetes history | 2.576 (0.988 ~ 6.717) | 0.053 |

**TABLE S4** (*Continued*) Univariable logistic regression of clinical variables and high ROS content.

| Variables | High ROS content | |
| --- | --- | --- |
|  | OR (95%CI) | *p* value |
| Hyperlipidemia history | 0.788 (0.353 ~ 1.756) | 0.560 |
| Smoking history | 1.433 (0.635 ~ 3.232) | 0.386 |
| Alcohol consumption | 3.800 (1.560 ~ 9.256) | 0.003^*^ |
| PBMC count (×10^9^/L) | 0.863 (0.545 ~ 1.366) | 0.529 |

ROS was grouped by the median, high ROS content ≥5.71.

Abbreviations: ROS, reactive oxygen species; BMI, body mass index; SBP, systolic blood pressure; DBP, diastolic blood pressure; HbA1c, glycated hemoglobin; FBG, fasting blood glucose; TC, total cholesterol; TG, triglyceride; LDL-C, low-density lipoprotein cholesterol; HDL-C, high-density lipoprotein cholesterol; Hcys, homocysteine; UA, serum uric acid; hs-CRP, hypersensitive C-reactive protein; FIB, fibrinogen; PBMC, peripheral blood mononuclear cells; OR, odds ratio; CI, confidence intervals.

^*^Statistically significant differences (*p* value <0.05).

**TABLE S5** Univariable logistic regression of clinical variables and 90-day unfavorable functional outcome.

| Variables | 90-day unfavorable functional outcome | |
| --- | --- | --- |
|  | OR (95%CI) | *p* value |
| Age (years) | 1.000 (0.960 ~ 1.041) | 0.994 |
| Male | 1.531 (0.540 ~ 4.339) | 0.423 |
| NIHSS (point) | 2.001 (1.482 ~ 2.702) | <0.001^*^ |
| BMI (kg/m^2^) | 1.093 (0.977 ~ 1.222) | 0.122 |
| SBP (mmHg) | 1.014 (0.992 ~ 1.036) | 0.212 |
| DBP (mmHg) | 1.009 (0.973 ~ 1.047) | 0.629 |
| HbA1c (%) | 1.005 (0.745 ~ 1.356) | 0.975 |
| FBG (mmol/L) | 1.050 (0.871 ~ 1.266) | 0.606 |
| TC (mmol/L) | 1.422 (1.001 ~ 2.021) | 0.050 |
| TG (mmol/L) | 1.344 (0.797 ~ 2.268) | 0.268 |
| LDL-C (mmol/L) | 1.661 (1.073 ~ 2.570) | 0.023^*^ |
| HDL-C (mmol/L) | 0.243 (0.042 ~ 1.404) | 0.114 |
| Hcys (μmol/L) | 0.983 (0.934 ~ 1.034) | 0.503 |
| UA (mmol/L) | 1.002 (0.997 ~ 1.007) | 0.439 |
| hs-CRP (mg/L) | 1.040 (0.964 ~ 1.121) | 0.311 |
| FIB (g/L) | 1.126 (0.694 ~ 1.827) | 0.630 |
| D-Dimer (mmol/L) | 1.145 (0.907 ~ 1.446) | 0.255 |
| Hypertension history | 1.824 (0.608 ~ 5.473) | 0.284 |

**TABLE S5** (*Continued*) Univariable logistic regression of clinical variables and 90-day unfavorable functional outcome.

| Variables | 90-day unfavorable functional outcome | |
| --- | --- | --- |
|  | OR (95%CI) | *p* value |
| Diabetes history | 0.711 (0.234 ~ 2.158) | 0.546 |
| Hyperlipidemia history | 0.745 (0.290 ~ 1.918) | 0.542 |
| Smoking | 1.108 (0.435 ~ 2.826) | 0.829 |
| Alcohol consumption | 1.108 (0.427 ~ 2.877) | 0.834 |
| PBMC count (×10^9^/L) | 1.223 (0.722 ~ 2.071) | 0.454 |

90-day unfavorable functional outcome was demined as mRS >2.

Abbreviations: NIHSS, National Institute of Health Stroke Scale; BMI, body mass index; SBP, systolic blood pressure; DBP, diastolic blood pressure; HbA1c, glycated hemoglobin; FBG, fasting blood glucose; TC, total cholesterol; TG, triglyceride; LDL-C, low-density lipoprotein cholesterol; HDL-C, high-density lipoprotein cholesterol; Hcys, homocysteine; UA, serum uric acid; hs-CRP, hypersensitive C-reactive protein; FIB, fibrinogen; PBMC, peripheral blood mononuclear cells; OR, odds ratio; CI, confidence intervals.

^*^Statistically significant differences (*p* value <0.05).
